# Serum levels of soluble urokinase plasminogen activator receptor (suPAR) predict outcome after resection of colorectal liver metastases

**DOI:** 10.18632/oncotarget.25471

**Published:** 2018-06-05

**Authors:** Sven H. Loosen, Frank Tacke, Marcel Binnebosel, Catherine Leyh, Mihael Vucur, Florian Heitkamp, Wenzel Schoening, Tom F. Ulmer, Patrick H. Alizai, Christian Trautwein, Alexander Koch, Thomas Longerich, Christoph Roderburg, Ulf P. Neumann, Tom Luedde

**Affiliations:** ^1^ Department of Medicine III, University Hospital RWTH Aachen, 52074 Aachen, Germany; ^2^ Department of Visceral and Transplantation Surgery, University Hospital RWTH Aachen, 52074 Aachen, Germany; ^3^ Division of Gastroenterology, Hepatology and Hepatobiliary Oncology, University Hospital RWTH Aachen, 52074 Aachen, Germany; ^4^ Institute of Pathology, University Hospital RWTH Aachen, 52074 Aachen, Germany; ^5^ Division of Translational Gastrointestinal Pathology, Institute of Pathology, University Hospital Heidelberg, 69120 Heidelberg, Germany; ^6^ Department of Surgery, Maastricht University Medical Centre (MUMC), Maastricht, The Netherlands

**Keywords:** cancer, biomarker, acute kidney injury, prognosis, CEA

## Abstract

**Background:**

In colorectal cancer (CRC), the liver is the most common site of metastasis. Surgical resection represents the standard therapy for patients with colorectal liver metastases (CRLM). However, 5-year survival rates after resection do not exceed 50%, and despite existing preoperative stratification algorithms it is still debated which patients benefit most from surgical treatment. The soluble urokinase plasminogen activator receptor (suPAR) has recently evolved as a promising biomarker for distinct clinical conditions. Here, we examined a potential role of suPAR as a biomarker in patients undergoing resection of CRLM.

**Results:**

Correlating with upregulated uPAR tissue expression in resected metastases, serum concentrations of suPAR were significantly elevated in CRLM patients compared to healthy controls. Importantly, patients with preoperative suPAR serum levels above the identified ideal cut-off value of 4.83 ng/ml showed a significantly reduced overall survival after resection of CRLM, both in right- and left-sided primary CRC. Moreover, multivariate Cox regression analysis revealed preoperative suPAR serum levels as a prognostic factor for mortality. Additionally, elevated preoperative suPAR but not creatinine levels were a predictor of acute kidney injury (AKI) after CRLM resection, correlating with a longer postoperative hospitalization.

**Conclusion:**

SuPAR represents a promising novel biomarker in CRLM patients that might help to guide preoperative treatment decisions regarding patients’ outcome and to identify patients particularly susceptible to AKI.

**Methods:**

Expression levels of uPAR were analyzed in CRLM tissue using RT-PCR and immunohistochemistry. SuPAR serum levels were measured by ELISA in 104 CRC patients undergoing hepatic resection for CRLM and 50 healthy controls.

## INTRODUCTION

Colorectal cancer (CRC) represents the third most common type of cancer worldwide and has remained one of the leading causes of cancer-related death to date [1]. Although disease occurrence is constantly decreasing in western countries, the overall incidence rate of CRC is still about 35 cases per 100,000 population for women and 50 cases per 100,000 population for men [2].

The lifetime incidence of colorectal liver metastases (CRLM) in CRC patients is approximately 50% [3]. For resectable CRLM patients, surgical removal of the liver metastases has evolved as the standard curative therapeutic approach [4]. However, only about 10–25 % of patients with CRLM qualify for surgical treatment, and around 65% of patients develop disease recurrence within three years [5]. The overall 5-year survival rate after surgical resection varies between 25 and 58% [6] compared to less than 1% for patients with advanced stage disease undergoing systemic chemotherapy [5], corroborating the medical benefit of a surgical treatment. Nevertheless, surgical resection can be associated with both local and systemic postoperative complications, which are associated with an unfavorable prognosis after surgery [7]. Among these, acute kidney injury (AKI) is a common postoperative complication following partial hepatectomy, which occurs in 6.2 to 12.1% of cases and results in an increased postoperative mortality [8]. Importantly, despite the existence of prognostic algorithms such as the FONG-score [9] and other preoperative assessment strategies (including laboratory parameters, imaging techniques and the clinical performance status), it is still not fully understood which patients actually benefit from surgical resection of CRLM in terms of overall survival (OS).

The soluble urokinase plasminogen activator receptor (suPAR) has recently emerged as novel biomarker in different clinical settings [10, 11]. suPAR represents the cleavage product of the membrane plasminogen activator receptor (uPAR), which is expressed on the surface of a variety of cells including endothelial or immune cells and is involved in the regulation of cell adhesion and migration [11]. Elevated levels of suPAR were described for a variety of clinical conditions including systemic inflammation as well as malignant diseases and have been suggested as a prognostic marker in gastric cancer patients [10, 12].

In this study, we aim to evaluate a potential prognostic role of circulating suPAR in a cohort of 104 metastatic CRC patients undergoing resection of CRLM at our tertiary referral hospital.

## RESULTS

### uPAR tissue expression levels are upregulated in colorectal liver metastasis

**Figure 1:**
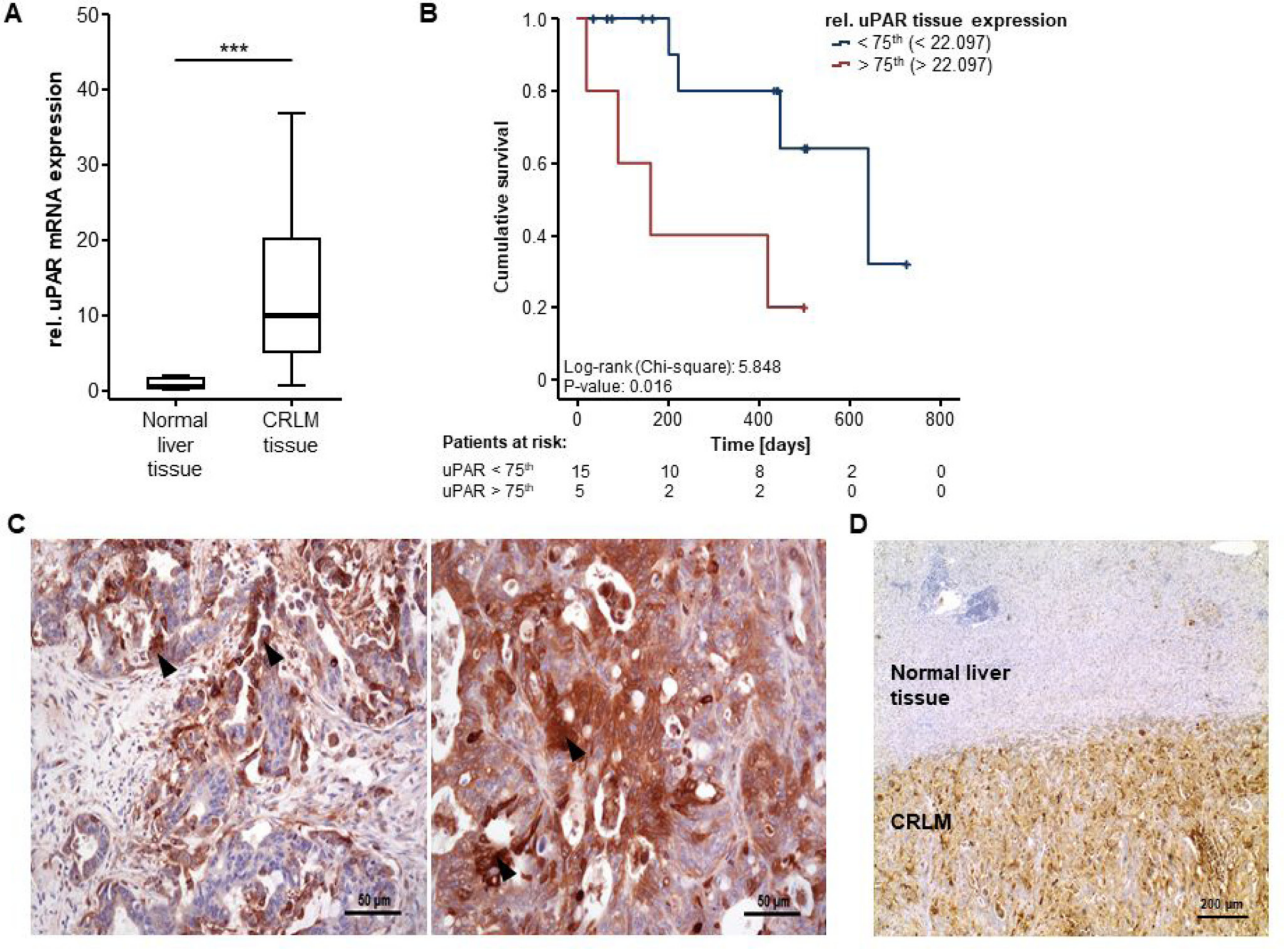
uPAR is overexpressed in colorectal liver metastases (**A**) uPAR mRNA expression is significantly upregulated in CRLM tissue samples compared to normal liver tissue (*U*-Test, *p* < 0.001). (**B**) uPAR tissue expression levels are associated with patients’ survival after resection of CRLM (log-rank test, *p* = 0.016). (**C**) Immunohistochemistry staining reveals a strong uPAR expression in tumour cells of CRLM (black arrow heads, 200-fold magnification). (**D**) In contrast to CRLM tissue, normal liver tissue shows only a very weak uPAR staining (40-fold magnification).

We first analyzed if CRLM display an upregulation of uPAR, the most common membrane bound source of circulating suPAR. Here, we found that uPAR mRNA expression is significantly elevated in 20 CRLM tissue samples (median: 10.10, IQR: 5.06–22.10) compared to normal liver tissue (median: 0.67, IQR: 0.32–1.78; Figure 1A). Interestingly, uPAR tissue expression levels were associated with patients’ survival after resection of CRLM, with a significantly impaired long-term survival for patients with high uPAR tumour expression (above the 75th percentile, Figure 1B). Moreover, immunohistochemical staining revealed a strong uPAR expression in CRLM that predominately occurs in tumour cells (black arrow heads, Figure 1C). In contrast, normal liver tissue only shows a very weak uPAR expression (Figure 1D).

### Serum levels of suPAR are elevated in patients with colorectal liver metastasis

**Figure 2:**
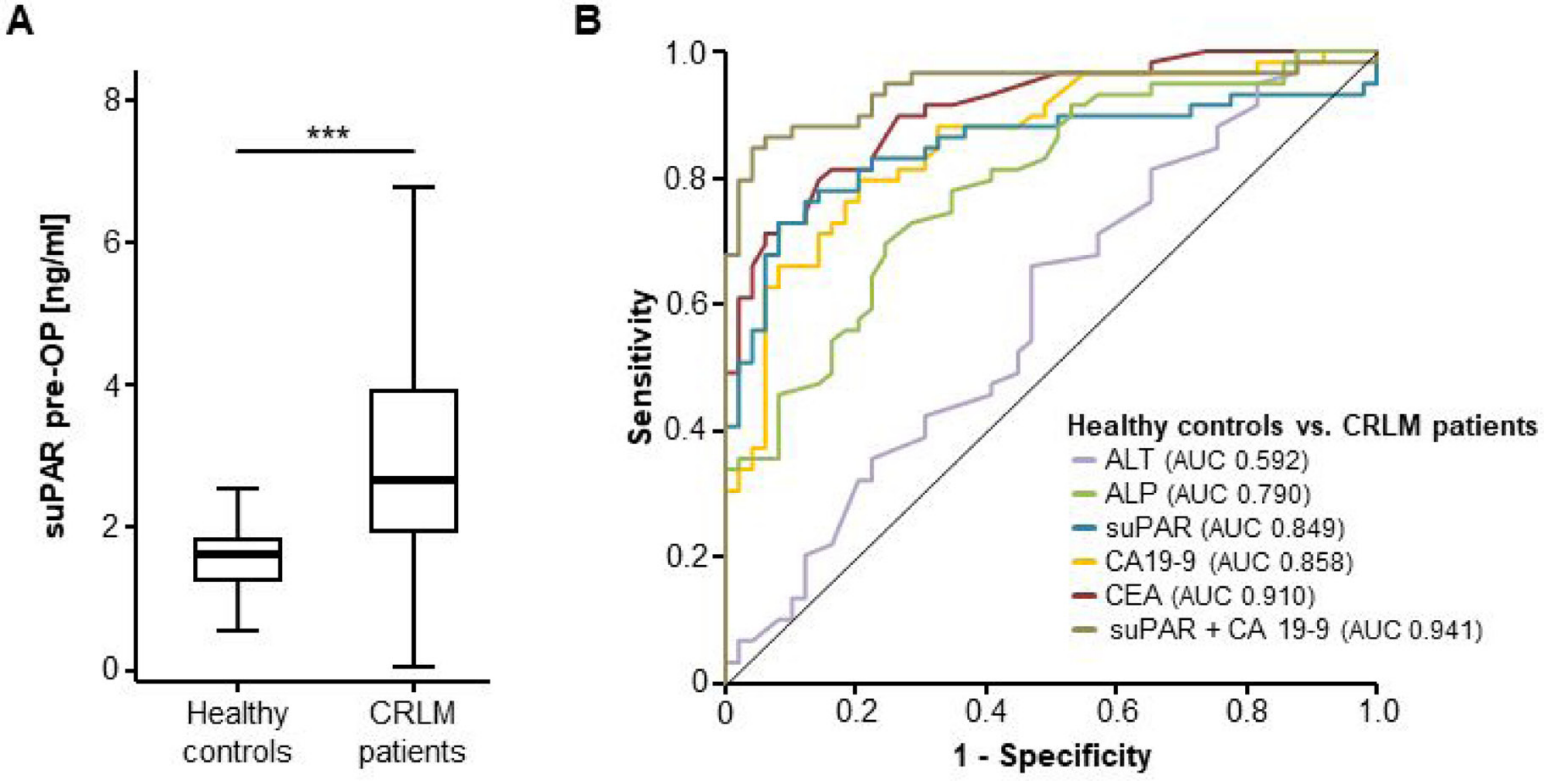
SuPAR serum levels are elevated in patients with colorectal liver metastasis (**A**) Serum levels of suPAR are significantly elevated in patients with CRLM compared to healthy controls (*U*-Test, *p* < 0.001). (**B**) ROC curve analysis reveals a similar diagnostic power of circulating suPAR levels compared to CEA and CA19-9 while ALT and ALP levels are unsuitable for the differentiation between CRLM patients and healthy controls. The combination of CEA and suPAR has the highest diagnostic power.

Based on the clear data on an upregulated uPAR expression in CRLM, we next compared preoperative levels of circulating suPAR between patients with CRLM and healthy controls (patient characteristics and laboratory parameters are displayed in Tables 1 and 2). In accordance with the uPAR tissue expression data, CRLM patients showed significantly elevated serum levels of suPAR compared to healthy control samples (Figure 2A). ROC curve analysis revealed an AUC of 0.849 for the discrimination between CRLM patients and healthy controls (Figure 2B). At an ideal cut-off value of 2.18 ng/ml, the diagnostic sensitivity and specificity was 72.9% and 91.8%, respectively. Moreover, when compared to classical tumour markers for CRC in this context, circulating levels of suPAR showed an only slightly lower diagnostic power than CEA (AUC 0.910) or CA19-9 (AUC 0.858) but was higher than standard markers of liver damage or cholestasis such as ALT (AUC 0.592) or ALP (AUC 0.790) (Figure 2B). Importantly, the combination of CEA and suPAR revealed the highest diagnostic power with an AUC of 0.941 (Figure 2B).

**Table 1:** Characteristics of study population

	Study cohort
Patients with CRLM	104
Healthy controls	50
Sex [%]: male-female	67.0–33.0
Age [years, median and range]	63 [25–85]
BMI [kg/m^2^, median and range]	25.42 [18.2–38.74]
Primary CRC characteristics [%]: G2-G3 right-sided - left-sided *KRAS* wt - *KRAS* mut	85.3–14.7 19.6–80.4 55.3–44.7
Largest size of CRLM [cm, median and range]	3.0 [0.5–14.0]
Clinical performance status [%]: ECOG 0-1-2-3	66.3-31.7–1.0-1.0
Synchronous resection vs. metachronous resection [%]	13.6–86.4
Postoperative AKI [%]: Yes-No	6.8–93.2
Deceased during follow-up [%]: Yes-No	37.1–62.9

**Table 2:** Serum levels of laboratory markers

	CRLM patients median [range], number of analyzed patients	Healthy controls median [range], number of analyzed patients
suPAR pre-OP [ng/ml]	2.67 [0.57–24.96], *n* = 104	1.62 [0.56–2.91] *n* = 50
suPAR post-OP [ng/ml]	3.49 [0.28–24.96], *n* = 82	–
CEA [µg/l]	7.35 [0.30–2703], *n* = 104	1.25 [0.3–6.3], *n* = 50
CA 19-9 [U/ml]	22.05 [0.6–4708], *n* = 48	5.4 [0–44.1], *n* = 50
WBC [cells/nl]	6.4 [1.9–18.5], *n* = 103	–
CRP [mg/l]	3.2 [0–120.6], *n* = 99	–
AST [U/l]	28.5 [2.1–399], *n* = 104	28 [20–78], *n* = 50
ALT [U/l]	23.5 [11–180], *n* = 64	20 [5–82], *n* = 50
GGT [U/l]	51 [10–1708], *n* = 99	17 [8–120], *n* = 50
ALP [U/l]	90 [41–479], *n* = 96	65 [36–102], *n* = 50
Bilirubin [mg/dl]	0.52 [0.12–1.29], *n* = 103	0.41 [0.1–1.46], *n* = 50
Creatinine [mg/dl]	0.84 [0.46–1.4], *n* = 104	–
Sodium [mmol/l]	140 [128–146], *n* = 102	–
Potassium [mmol/l]	4.3 [2.6–5.9], *n* = 104	–
Hemoglobin [g/l]	13.3 [8.2–16.9], *n* = 103	–
Platelets [cells/nl]	234.5 [102–782], *n* = 102	–

Abbreviations: suPAR: soluble urokinase plasminogen activator receptor, CEA: carcinoembryonic antigen, CA 19-9: carbohydrate-Antigen 19-9, WBC: white blood cell count, CRP: C-reactive protein, AST: aspartate transaminase, ALT: alanine transaminase, GGT: γ-Glutamyl transpeptidase, ALP: alkaline phosphatase.

We next investigated if suPAR serum levels might reflect specific disease characteristic such as the size of CRLM, the tumour grading or the localization (right- vs. left-sided) of the initial CRC. While suPAR serum levels showed a significant correlation with the largest diameter of CRLM (Supplementary Figure 1A), no differences in circulating suPAR levels became apparent when comparing moderately (G2) or poorly (G3) differentiated tumors (Supplementary Figure 1B) or left- vs. right-sided primary CRC (Supplementary Figure 1C). Similarly, serum suPAR levels did not differ between patients with a *KRAS* wild type or *KRAS* mutated CRC (Supplementary Figure 1D) as well as patients with different ECOG performance status (Supplementary Figure 1E). Finally, suPAR serum levels were unaltered between male and female patients (Supplementary Figure 1F).

### Elevated levels of circulating suPAR are associated with a reduced overall survival after resection of colorectal liver metastases

**Figure 3:**
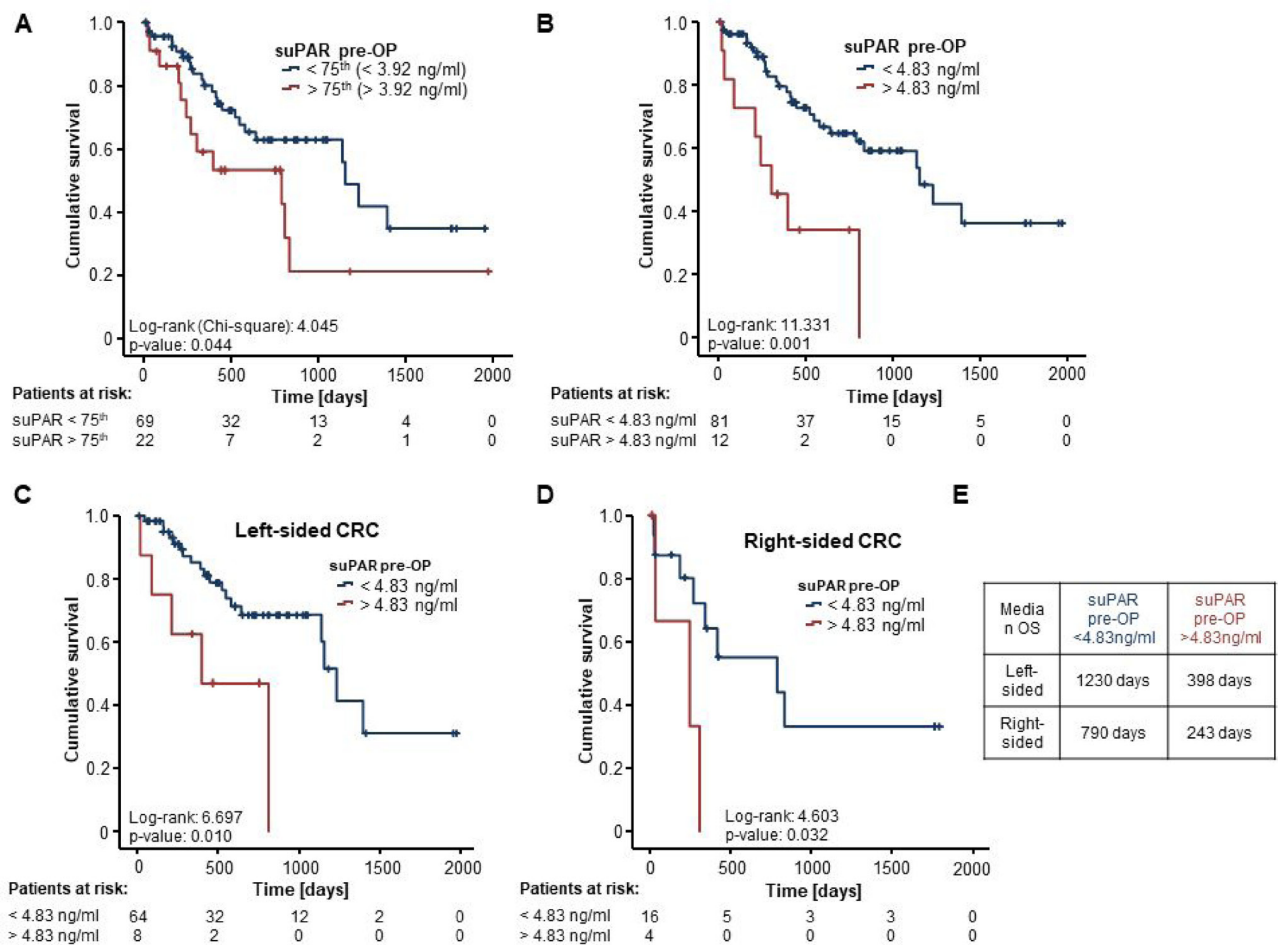
Elevated levels of circulating suPAR are associated with a reduced overall survival after resection of colorectal liver metastases (**A**) Patients with high preoperative suPAR serum levels (>75th percentile) show a significantly impaired overall survival (OS) compared to patients with low suPAR levels (log-rank test, *p* = 0.044). (**B**) When applying our ideal cut-off value of 4.83 ng/ml, patients with initial suPAR serum above the cut-off show a strikingly reduced OS (median OS: 304 days) compared to patients with serum suPAR levels below this cut-off (median OS: 1154 days) (log-rank test, *p* = 0.001). (**C** and **D**) Kaplan-Meier curve analyses reveal that both left- and right-sided primary CRC patients with high initial suPAR serum levels above our cut-off value display a significantly reduced OS compared to patients with low initial suPAR serum concentrations (log-rank test, left: *p* = 0.010, right: *p* = 0.032). (**E**) The median OS is highest in patients with left-sided primary CRC and suPAR serum levels below the cut-off value whereas patients with right-sided disease and high suPAR levels have the worse postoperative outcome.

We next evaluated the potential prognostic role of preoperative suPAR serum levels in our cohort of CRLM patients. We therefore subdivided our cohort into two groups of patients with either high or low initial suPAR measurements (above or below the 75th percentile). Interestingly, Kaplan-Meier curve analysis showed a significantly impaired long-term survival for patients with high suPAR serum levels (above the 75th percentile) compared to patients with low suPAR levels (below the 75th percentile) (Figure 3A). To establish an ideal cut-off value for the discrimination between survivors and non-survivors, we used an established biometric software (see *Material and Methods* for detailed information) [13], which revealed an optimal cut-off suPAR value of 4.83 ng/ml. When applying this cut-off to our cohort of patients, the prognostic power of circulating suPAR levels was even superior, showing a strikingly reduced survival for patients with suPAR levels greater than 4.83 ng/ml (Figure 3B). As such, median survival of patients with initial suPAR levels below 4.83 ng/ml was 1154 days compared to 304 days for patients with suPAR levels above our ideal cut-off. Moreover, only patients with suPAR concentrations below our cut-off showed a long-term survival beyond 5 years while no patient with suPAR serum levels above the cut-off reached long-term survival.

To further explore these results on a potential prognostic role of suPAR serum levels, we next performed extensive uni- and multivariate Cox-regression analysis including various laboratory parameters such as tumor markers (CEA and CA19-9), markers of liver and kidney function (AST and creatinine), inflammatory parameters (leucocyte count and CRP) as well as clinical and pathological parameters (BMI, largest diameter of CRLM, location of primary CRC). In univariate analysis, preoperative suPAR levels above our ideal cut-off value turned out as a strong prognostic factor for OS (HR 3.680 [1.633–8293], *p* = 0.002). Moreover, in multivariate Cox-regression analysis including all parameters with a *p*-value < 0.250 in univariate testing, suPAR serum levels stood out as a prognostic marker for overall survival (HR 2.935 [1.088–7.961], *p* = 0.033, Table 3).

**Table 3:** Uni- and multivariate Cox-regression analyses for the prediction of patients’ outcome after tumour resection

	Univariate Cox-regression		Mulitvariate Cox-regression	
Parameter	*p*-value	Hazard-Ratio (95% CI)	*p*-value	Hazard-Ratio (95% CI)
suPAR (>4.83 ng/ml)	0.002	3.680 [1.633–8.293]	0.033	2.935 [1.088–7.916]
CEA	<0.001	1.001 [1.000–1.002]	0.013	1.001 [1.000–1.003]
CA19-9	0.011	1.000 [1.000–1.001]	0.967	1.000 [0.999–1.001]
CRP	0.012	1.015 [1.003–1.027]	0.369	1.009 [0.989–1.030]
Leukocyte count	0.017	1.171 [1.028–1.333]	0.436	1.065 [0.909–1.248]
Creatinine	0.822	0.831 [0.165–4.194]		
AST	0.044	1.004 [1.000–1.008]	0.336	1.003 [0.997–1.008]
BMI	0.580	1.019 [0.953–1.090]		
Largest diameter of CRLM	0.424	1.040 [0.945–1.144]		
Right- vs. left-sided primary CRC	0.146	1.708 [0.830–3.513]	0.080	2.119 [0.915–4.907]

Abbreviations: suPAR: soluble urokinase plasminogen activator receptor, CEA: carcinoembryonic antigen, CA19-9: carbohydrate antigen 19-9, CRP: C-reactive protein, AST: aspartate transaminase, BMI: Body-Mass-Index, CRLM: colorectal liver metastasis, CRC: colorectal cancer

Given recent data on the importance of the primary tumour localization in metastasized CRC patients [14–16], we separately analyzed the prognostic relevance of serum suPAR levels in patients with primary right- vs. left-sided CRC. When applying our previously established cut-off value of 4.83 ng/ml, Kaplan-Meier curve analysis revealed that both left- and right-sided primary CRC patients with high initial suPAR serum levels above our cut-off display a significantly reduced OS compared to patients with low initial suPAR serum concentrations (Figure 3C and 3D). Notably, the median OS was highest in patients with left-sided primary CRC and suPAR serum levels below our ideal cut-off value, whereas patients with right-sided disease and high suPAR levels had the worse postoperative outcome (Figure 3E). However, patients with right-sided CRC and low suPAR levels (below the ideal cut-off value) still had a better prognosis than patients with left-sided primary tumour and high suPAR serum levels (Figure 3E). Finally, we examined if serum levels of suPAR undergo longitudinal changes after surgical resection of CRLM. Postoperative serum samples were available for 82 patients and showed a significant increase in suPAR concentrations compared to the related preoperative serum samples (Supplementary Figure 2A). Moreover, spearman correlation analysis revealed a strong correlation between pre- and postoperative suPAR serum levels (*r* = 0.784, *p* < 0.001). While there was no significant differences in postoperative suPAR concentration between moderately and poorly differentiated tumors or *KRAS* mutated and non-mutated patients (Supplementary Figure 2B and 2C), CRLM patients with right-sided primary CRC showed significantly elevated suPAR levels compared to left-sided CRC patients (Supplementary Figure 2D). However, neither postoperative suPAR serum levels (Supplementary Figure 3A) nor the individual kinetics of suPAR serum concentrations before and after surgery (Supplementary Figure 3B) had an impact on the patients’ survival in Kaplan Meier-curve analysis. In line, Cox-regression analysis revealed that both postoperative suPAR levels (HR: 1.153 [0.972–1.367], *p* = 0.101) as well as the individual kinetic of suPAR (HR: 0.862 [0.385–1.929], *p* = 0.718), were unsuitable for the prediction of long-term survival. Together, our data show that preoperative but not postoperative suPAR serum concentrations represent a promising prognostic biomarker for patients undergoing surgical resection of liver metastases from left- and right-sided CRC.

### Preoperative levels of suPAR predict acute kidney injury after surgical resection of CRLM

**Figure 4:**
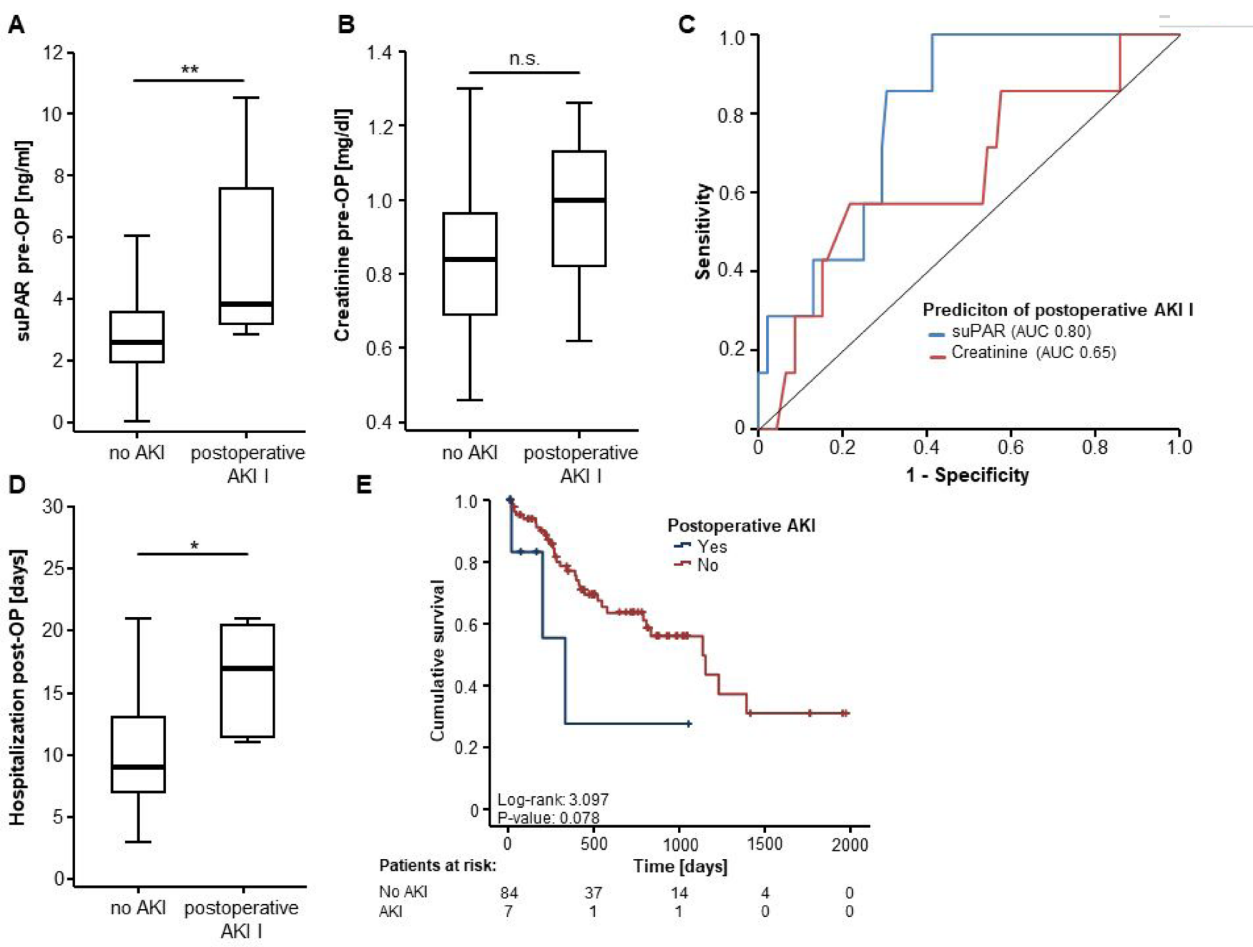
Preoperative levels of suPAR predict acute kidney injury after surgical resection of CRLM (**A**) Preoperative suPAR serum levels are significantly elevated in patients who develop acute kidney injury (AKI) after surgical resection of CRLM compared to non-AKI patients (*U*-Test, *p* = 0.009). (**B**) In contrast, preoperative creatinine levels are unaltered in AKI and non-AKI patients (*T*-Test, *p* = 0.137). (**C**) Serum levels of suPAR are superior to creatinine levels for the differentiation between patients with postoperative AKI and non-AKI patients. (**D**) The occurrence of AKI after CRLM resection is associated with a significantly longer postoperative duration of hospitalization (*U*-Test, *p* = 0.010). (**E**) Patients who present with postoperative AKI show a strong trend towards an impaired postoperative prognosis (log-rank test, *p* = 0.078).

To investigate a potential correlation between high suPAR levels and an impaired postoperative renal function in our cohort of patients, we next compared circulating levels of suPAR in patients that developed acute kidney injury (AKI) after resection of CRLM and patients who presented with a normal postoperative renal function. Interestingly, preoperative suPAR levels were significantly elevated in those patients who developed postoperative AKI I according to KDIGO criteria [17] (Figure 4A). In contrast, initial serum creatinine levels did not significantly differ between AKI and non-AKI patients (Figure 4B). In line, ROC curve analysis revealed a superior AUC value for serum suPAR levels for the discrimination between AKI and non-AKI patients compared to serum creatinine levels (AUC_suPAR_ 0.80 vs. AUC_Crea_ 0.65) (Figure 4C). At an optimal cut-off value of 2.82 ng/ml, serum levels of suPAR showed a sensitivity and specificity of 100% and 58.7% for the prediction of AKI. To further substantiate the predictive potential of preoperative suPAR levels, we next performed a univariate binary logistic regression analysis. Here, only preoperative suPAR serum levels (OR 1.256 [1.039–1.518], *p* = 0.018) but not serum creatinine levels (OR 14.429 [0.403–516.123], *p* = 0.144) stood out as predictor of postoperative AKI. However, the number of patients developing postoperative AKI I (*n* = 7) in our cohort was too small to perform sufficient multivariate regression analysis.

To evaluate, whether the occurrence of postoperative AKI had a direct impact on the clinical course of the patients, we subsequently compared the postoperative duration of hospitalization for patients that developed AKI after resection of CRLM and non-AKI patients. Interestingly, non-AKI patients were discharged significantly earlier from hospital after surgery compared to patients that developed AKI (median duration of hospitalization: 9 vs. 17 days), underlining the clinical relevance of AKI in this setting (Figure 4D). Moreover, Kaplan-Meier curve analysis revealed a strong trend (not significant) towards an impaired OS for patients developing AKI after surgical resection of CRLM (Figure 4E). Univariate Cox-regression for the impact of AKI on OS analysis showed a similar trend with an HR of 2.800 [0.845–9.278, *p* = 0.092].

## DISCUSSION

In contrast to other GI malignancies, the median overall survival of patients with metastasized CRC has constantly improved over the last decades [3]. Today, various multimodal therapeutic approaches can be offered to these patients, especially to those with liver-limited disease [3, 4]. As such, surgical therapy of CRLM is not only performed as a palliative option to reduce the hepatic tumour burden, but can also be performed in curative intent and result in long-term survival in up to 30% of cases [4]. However, new systemic treatment options were recently introduced into clinical algorithms for metastasized CRC patients, including e.g. specific antibodies or tyrosine kinase inhibitors, offering several lines of chemotherapy which can also lead to a considerably prolonged survival even without surgical resection [18, 19]. With all these options available, the individual decision in the interdisciplinary tumour board whether a CRLM patient should receive surgical resection or rather be enrolled in a conservative therapeutic approach is challenging. At present, the decision for or against surgical treatment is often based on the patient’s performance status and the technical resectability (including imaging techniques and the assessment of liver function), whereas e.g. aspects of tumour biology are less frequently considered [20]. Therefore, preoperatively available biomarkers could help to better characterize which patients would actually benefit from surgical resection of CRLM in terms of a personalized therapeutic approach.

Here, we show that suPAR represents a promising prognostic marker for patients undergoing resection of CRLM and might especially be useful to provide further information to the pivotal question whether or not a patient will reach long-term survival after resection. CRLM patients with preoperative suPAR serum levels above our defined ideal cut-off value of 4.83 ng/ml showed a strikingly impaired postoperative prognosis with a median overall survival (OS) of only 304 days. Importantly, none of these patients reached long-term survival (>3 years). On the opposite site, patients with initial suPAR serum levels below the cut-off value had a significantly better long term prognosis, with a median OS of 1154 days (see Figure 3). In line with recent evidence from large clinical trials [14], patients with metastases from right-sided primary CRC had a worse prognosis than those with left-sided in our cohort, but in both subgroups suPAR serum levels maintained their discriminative prognostic potential. Importantly, this prognostic function was independent of systemic inflammation, liver and kidney function, other CRC tumor markers such as CEA and CA19-9 as well as clinical features like patients’ BMI, the primary CRC tumor side or the tumor burden in multivariate Cox-regression analysis (see Table 3). However, further larger clinical studies are warranted to evaluate if suPAR serum levels might serve as a possible addition to existing preoperative stratification tools such as the FONG score [9].

SuPAR is the cleavage product of the membrane bound plasminogen activator receptor (uPAR), which is expressed on the surface of a variety of cells including endothelial and immune cells and has been associated with several clinical conditions such as systemic inflammation and cancer [10]. Nevertheless, the exact source of elevated serum suPAR levels in cancer patients including our cohort of CRLM patients is not fully elucidated. It was previously shown that both primary CRC and CRLM show a strong uPAR expression which predominately origins from infiltrating immune cells such as macrophages and neutrophils but also from malignant tumour cells and the stromal tissue [21, 22]. In line, we confirmed that uPAR expression is highly upregulated in CRLM patients from our cohort of patients with a predominant uPAR expression in the tumour cells (see Figure 1). Thus, it is conceivable that elevated suPAR serum levels originate from an increased shedding of uPAR in the cancerous tissue of colorectal liver metastasis, a process that has previously been suggested to mirror tumour immune activation [23]. Moreover, experimental data support a direct association between the tumour mass and circulating levels of suPAR [24]. The exact pathophysiological link between high suPAR levels and a poor prognosis is presently not fully understood. SuPAR has been functionally linked to a reduced PTEN expression in endothelial cells which leads to an increased angiogenesis, embodying a key step of cancer development [25]. Moreover, suPAR was shown to activate the PI3K/Akt-pathway, one of the most frequently occurring molecular aberration in colorectal carcinoma [25, 26]. Finally, uPAR has been described as promoter of cell adhesion and migration, representing essential processes in the development of cancer. Nevertheless, further molecular studies are warranted, e.g. using *uPAR*^*-/-*^ mice, to fully elucidate a potential tumorigenic function of suPAR [27].

Interestingly, our data provided evidence for a predictive role of suPAR serum levels for the occurrence of postoperative AKI after resection of CRLM. Postoperative AKI occurs in about 10% of cases following partial liver resection and has a fundamental impact on the postoperative morbidity, mortality and duration of hospitalization [8, 28]. However, the prediction of an impaired renal function after liver resection is challenging. Serum creatinine levels represent the standard marker for the assessment of acute and chronic renal failure but its predictive value for the occurrence of postoperative AKI is limited [29], highlighting the specific value that suPAR might play to preoperatively identify these patients. Serum suPAR levels might not only be valuable to decide whether or not a CRC patient should undergo surgical resection, but might also raise clinical attention and trigger specific measures to prevent postoperative AKI in this vulnerable subgroup of patients. Importantly, postoperative AKI was associated with a significantly prolonged duration of hospitalization and a strong trend towards an impaired OS in our cohort of patients (see Figure 4), corroborating the clinical relevance of this finding in terms of a preoperative risk stratification. The pathophysiological relation between elevated suPAR levels and impairment of renal function again is not fully clear at present. However, these data are in line with a previous large trial, showing an independent association between suPAR serum levels and the incidence of chronic kidney disease and an accelerated decline in the glomerular filtration rate in 3683 patients undergoing cardiac catheterization [30].

While our data suggest a role of circulating suPAR as a prognostic marker in patients undergoing surgical resection of CRLM, it remains unclear if serum suPAR levels might also have a predictive value in CRLM patients receiving different treatment modalities (e.g. systemic chemotherapy, transarterial chemoembolization (TACE) or radiofrequency ablation (RFA)). Thus, further studies are warranted to not only confirm its prognostic role in the context of CRLM resection but also to assess if suPAR could support future biomarker-driven clinical decision algorithms in the multimodal treatment of metastasized colorectal cancer.

## MATERIALS AND METHODS

### Study design and patient characteristics

This observational cohort study was designed to evaluate suPAR as a serum marker in patients undergoing resection of colorectal liver metastases (CRLM). A total of 104 patients admitted to the Department of visceral and transplantation surgery at the University Hospital RWTH Aachen for surgical resection of CRLM were prospectively recruited between 2011 and 2016 and enrolled into this study (detailed patient characteristics are given in Table 1). Patients that died during or immediately after surgery on the intensive care unit (<14 days) were not included into the study. Serum samples were collected prior to surgery and 6–7 days after tumour resection. As a control population we analyzed 50 healthy, cancer-free blood donors with normal values for blood counts, C-reactive protein and liver function. The occurrence of acute kidney injury (AKI) stage I in the first 48 hours after surgery was assessed, as defined according to the current KDIGO criteria [17]. The study protocol was approved by the local ethics committee and conducted in accordance with the ethical standards laid down in the Declaration of Helsinki (ethics committee of the University Hospital Aachen, RWTH University, Aachen, Germany). Written informed consent was obtained from the patient or the appointed legal guardian.

### Measurement of serum suPAR levels

SuPAR serum concentrations were analyzed using a commercial enzyme-linked immunosorbent assay (ELISA) according to the manufacturer’s instructions (Nr. A001, suPARnostic, ViroGates, Birkerød, Denmark). Evaluation of the ELISA absorbance values and calculation of the serum concentration were performed using a 4 Parameter Logistic (4PL) nonlinear regression model. Other standard laboratory markers were measured in the laboratory center for blood analysis at University Hospital RWTH Aachen. Circulating levels of serum tumour markers (CEA and CA19-9) were analyzed with an electrochemiluminescence immunoassay (ECLIA) using the Cobas 8000 e602 modular analyzer series (Hoffmann-La Roche AG, Basel, Switzerland). Standard hematological and clinical chemistry parameters were measured using the Sysmex XN9000 (Sysmex GmbH, Norderstedt, Germany) and the Cobas 8000 c701 (Hoffmann-La Roche AG, Basel, Switzerland).

### Semi-quantitative reverse transcriptase PCR

RNA isolation from tissue samples, cDNA synthesis and semi-quantitative reverse transcriptase PCR was performed as recently described in detail according to the MIQE guidelines [31]. Following primers for uPAR were used: For-uPAR: 5′-TGTAAGACCAACGGGGATTGC-3′, Rev-uPAR: 5′-AGCCAGTCCGATAGCCAGG-3′.

### Immunohistochemistry

For rehydration and antigen retrieval, 3-micron sections of formalin-fixed, paraffin-embedded tissue were obtained and treated using the PT-Link module (DAKO, Glostrup, Denmark) at pH 9.0. To block unspecific background staining slides were treated with hydrogen peroxide and protein block solution (both DAKO). After washing, the primary Anti-uPA Receptor antibody (1:1000, ab218106, Cambridge, UK) was incubated for 60 min at RT. Visualization was performed using Envision Flex kit (DAKO) according to the manufacturer´s instructions. After counterstain with haematoxylin, sections were dehydrated and cover slipped.

### Statistical analysis

Serum data are given as median and range to reflect the skewed distribution of analyses on human samples. Kolmogorov-Smirnov- and Shapiro-Wilk-Test were used to test for normal distribution. Non-parametric data were compared using the Mann–Whitney-*U*-Test (two-sided). Parametric data were compared using the Student’s-*t*-Test (two-sided). Box plot graphics display a statistical summary of the median, quartiles and ranges. ROC curves were generated by plotting sensitivity against 1-specificity. The optimal cut-off values for ROC curves were established using the Youden-Index (YI = sensitivity + specificity - 1). The predictive value of suPAR with respect to AKI was tested using a binary logistic regression model. The odds ratio (OR) and the 95% confidence interval are displayed. Correlation analyses were performed using the Spearman correlation tests. Kaplan-Meier curves were plotted to display the impact on survival. The Log-rank test was used to test for differences between subgroups in Kaplan-Meier curve analysis. The optimal cut-off value for the identification of patients with an impaired OS was established using a recently published biometric software, which fits Cox proportional hazard models to the dichotomized survival status (*survivors:* patients that did not decease during follow-up vs. *non-survivors*: patients who died during follow-up) and the survival variable (survival time until event/censoring). The optimal cut-off value is then defined as the suPAR serum level with the most significant split of groups in log-rank testing. [13]. The prognostic value of variables was further tested by univariate and multivariate analysis in the Cox regression model. Inclusion criterion for multivariate testing was a *p*-value < 0.25 in univariate analysis. The hazard ratio (HR) and the 95% confidence interval are displayed. All statistical analyses were performed with SPSS 23 (SPSS, Chicago, IL, USA). A *p*-value of < 0.05 was considered statistically significant (^*^p < 0.05; ^**^p < 0.01; ^***^p < 0.001)

## SUPPLEMENTARY MATERIALS FIGURES



## Abbreviations

ALP: alkaline phosphatase
ALT: alanine transaminase
AST: aspartate transaminase
BMI: Body Mass Index
CA19-9: Carbohydrate antigen 19-9
CEA: Carcinoembryonic antigen
CRC: Colorectal cancer
CRLM: Colorectal liver metastasis
CRP: C-reactive protein
ECLIA: Electrochemiluminescence Immunoassay
GGT: γ-Glutamyl transpeptidase
HR: Hazard ratio
IQR: Interquartile range
OR: Odds ratio
OS: Overall survival
RFA: Radiofrequency ablation
suPAR: Soluble urokinase plasminogen activator receptor
TACE: Transarterial chemoembolization
uPAR: Urokinase receptor
WBC: White blood cell count (leucocyte count)
