# Supplementary material for: Serum levels of soluble urokinase plasminogen activator receptor (suPAR) predict outcome after resection of colorectal liver metastases
Source: Oncotarget. 2018 Jun 5;9(43):27027–38. doi: 10.18632/oncotarget.25471 (PMC6007468; doi:10.18632/oncotarget.25471)
Supplement: Supplementary file 1 [file oncotarget-09-27027-s001.pdf]

Serum levels of soluble urokinase plasminogen activator receptor (suPAR) predict outcome after resection of colorectal liver metastases

SUPPLEMENTARY MATERIALS

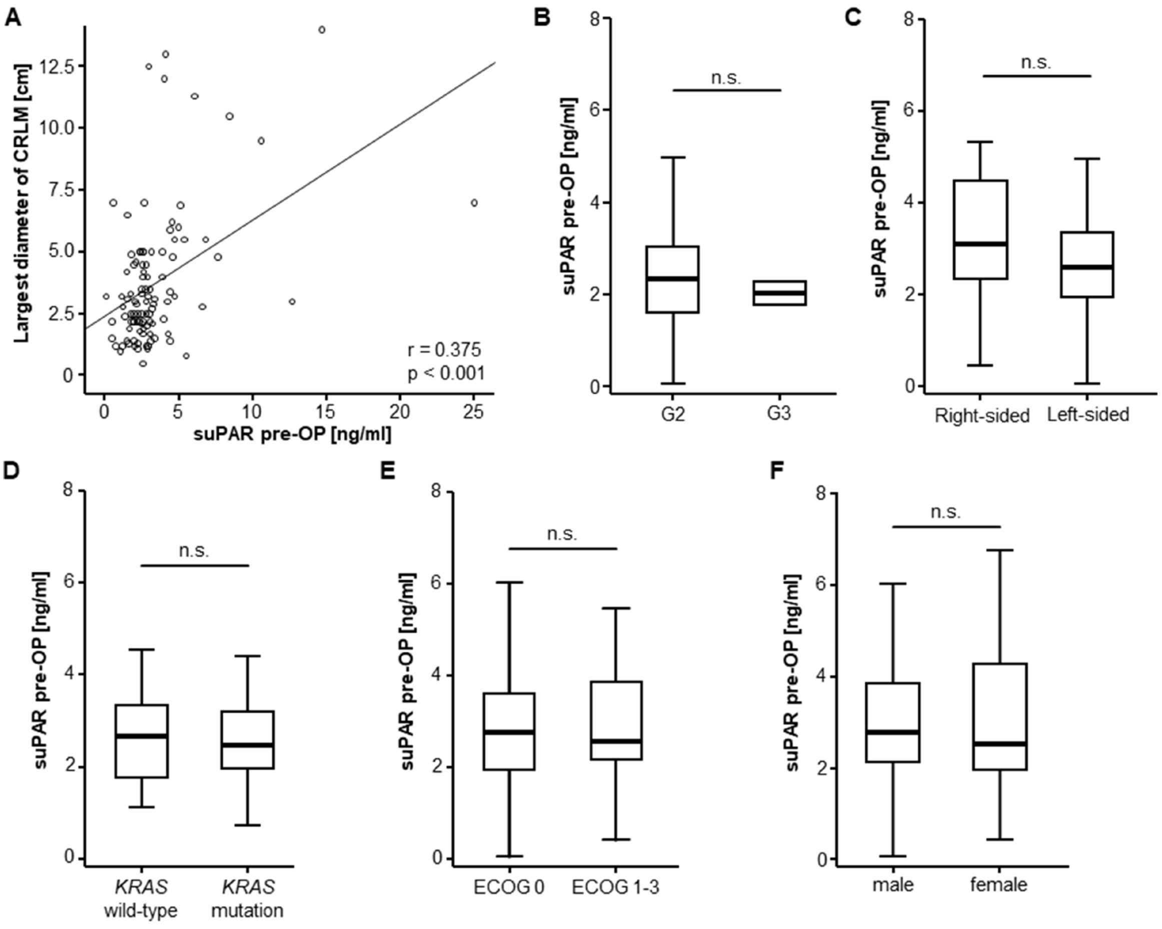

Supplementary Figure 1: Serum levels of suPAR and disease characteristics. (**A**) Initial suPAR serum levels significantly correlate with the largest diameter of CRLM (spearman correlation, $r = 0.375$, $p < 0.001$). The tumour grading (**B**, *U*-Test, $p = 0.508$), the localization of the primary CRC (**C**, *U*-Test, $p = 0.124$) or the *KRAS* mutation status (**D**, *U*-Test, $p = 0.676$) do not influence serum levels of circulating suPAR. Circulating suPAR levels do not reflect the patients' ECOG performance status (**E**, *U*-Test, $p = 0.618$) or sex (**F**, *U*-Test, $p = 0.565$).

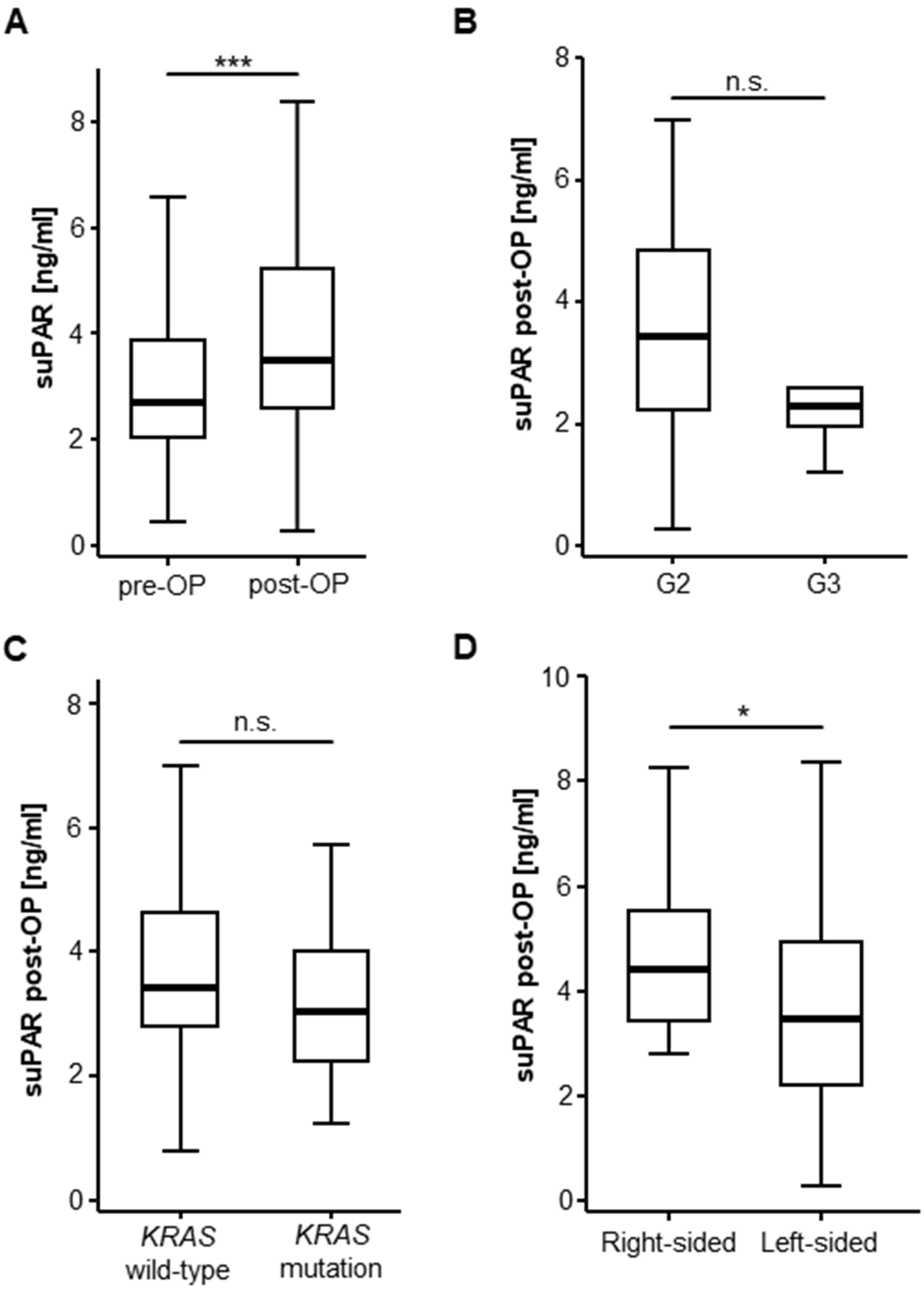

Supplementary Figure 2: Serum levels of suPAR increase after surgery. (**A**) Postoperative suPAR serum levels are significantly higher compared to preoperative values (Wilcoxon signed-rank test, $p < 0.001$). The tumour grading (**B**, *T*-Test, $p = 0.211$) and the *KRAS* mutation status (**C**, *T*-Test, $p = 0.290$) do not influence serum levels of circulating preoperative suPAR. (**D**) CRLM patients with right-sided primary CRC show significantly elevated suPAR levels compared to left-sided CRC patients (*U*-Test, $p = 0.039$).

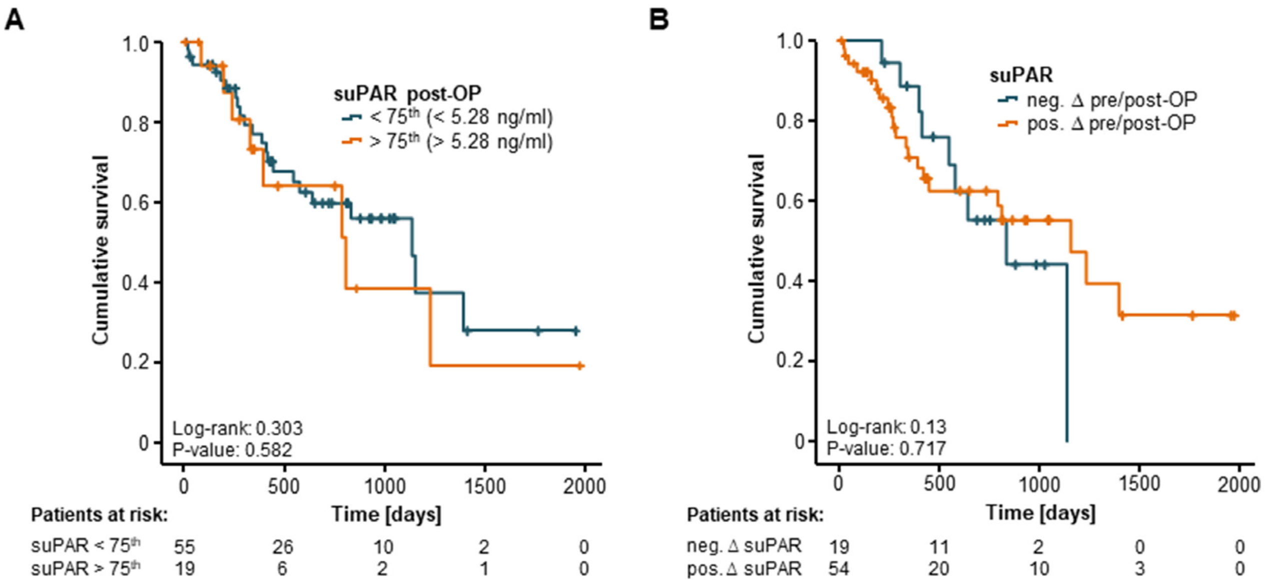

Supplementary Figure 3: Postoperative serum suPAR levels or longitudinal changes of circulating suPAR are unsuitable for the prediction of overall survival. (**A**) Patients with high postoperative suPAR serum levels (>75th percentile) do not have a significantly impaired overall survival (OS) compared to patients with low suPAR levels (log-rank test, $p = 0.582$). (**B**) Longitudinal changes of circulating suPAR levels before and after surgery do not reflect patients' OS (log-rank test, $p = 0.717$) .
